# Fruit scent as an honest signal for fruit quality

**DOI:** 10.1186/s12862-022-02064-z

**Published:** 2022-11-30

**Authors:** Omer Nevo, Kim Valenta, Annabella Helman, Jörg U. Ganzhorn, Manfred Ayasse

**Affiliations:** 1 German Centre for Integrative Biodiversity Research (iDiv) Halle-Jena-Leipzig, Leipzig, Germany; 2 Institute of Biodiversity, Friedrich Schiller University Jena, Jena, Germany; 3 Institute of Evolutionary Ecology and Conservation Genomics, Ulm University, Ulm, Germany; 4 Department of Anthropology, University of Florida, Gainesville, FL USA; 5 Department of Evolutionary Anthropology, Duke University, Durham, NC USA; 6 Animal Ecology and Conservation, University of Hamburg, Hamburg, Germany

**Keywords:** Animal–plant communications, Coevolution, Frugivory, Honest signaling, Seed dispersal

## Abstract

**Background:**

Fleshy fruits evolved to be attractive to seed dispersers through various signals such as color and scent. Signals can evolve through different trajectories and have various degrees of reliability. The strongest substrate on which reliable signals can evolve is when there is an inherent link between signal and reward, rendering cheating costly or impossible. It was recently proposed that aliphatic esters in fruit scent may be predictive of sugar content due to their synthesis from products of sugar fermentation. We test this hypothesis on a case study of wild fig species (*Ficus tiliifolia*) from Madagascar, which relies on seed dispersal by lemurs.

**Results:**

We found a strong positive correlation between signal (esters) and reward (sugar). We also found that non-esters, including direct fermentation products, in fruit scent do not indicate sugar levels, which implies that this relationship is not simply a product of fruit maturation wherein more mature fruits emit more scent and contain more sugar.

**Conclusions:**

While based on a single taxon, these results strongly support the hypothesis that a biochemical link between ester synthesis and sugar may render the ester fraction of fruit scent an honest signal for fruit quality, with consequences for animal sensory and feeding ecology, and the evolution of plants in the context of seed dispersal.

**Supplementary Information:**

The online version contains supplementary material available at 10.1186/s12862-022-02064-z.

## Background

Fleshy fruits have evolved to attract fruit-eating animals to consume them and thus facilitate seed dispersal [1]. In addition to nutrient rewards, various fruit traits such as size, color, taste, and scent/taste have evolved in response to the behavior and preference of seed dispersers [2]. Color [3–5] and scent [6–11], in particular, are seen as adaptations whose function is not to reward animals directly, but rather to increase the detectability of fruits and thus increase the probability of seed dispersal. Moreover, in some cases a connection between these attractants and fruit nutrient content has been documented. Variance in fruit coloration has been associated with the presence of macronutrients like sugar or fat across various species [12–17]. Recently, it was also suggested that variation in the chemical composition of fruit scent across species is associated with differences in sugar content [18], and that animals with high olfactory capacities like elephants are capable of detecting fruit sugar levels based on scent alone [19]. Similar relationships between signal and reward have been suggested to be involved in pollinator-flower interactions [20].

The vast majority of studies linking nutrient rewards to plant attractants focused on variation across plant species, e.g. showing that species whose fruits tend to bear certain colors or emit certain chemicals are on average more likely to be richer in certain nutrients. Variation within species is seldom studied, despite its high importance: first, it may facilitate the association between signal and reward and thus also help animals learn to identify more nutritious species. Second, intraspecific variation is a precondition for selection and adaptation. Finally, combining these two points, intraspecific variation signals and rewards would render the link be of high relevance to animals, thus exerting selection pressures on fruits. Competition between animal group members for high-quality fruit can be strong and it has been shown that individuals better capable of exploiting fruit signals like a conspicuous visual display have higher fruit intake [21]. Thus, signals that reliably predict fruit quality within species would allow animals to identify high-quality individuals and fruits, and given the benefit to seed dispersers can also be under positive selection in plants. This is exacerbated by strong within-species competition for seed dispersers [22] that may exert a selective pressure on plants to signal their quality. Yet to our knowledge, only one extant study tested whether variance in visual cues is associated with signals *within* species [23]. Another recent study also linked olfactory cues in marula fruits (*Sclerocaya birrea*) with their nutrient content [19], though this study focused on animal behavior, and therefore only sampled a limited number of fruits from a single tree.

Here, we test the hypothesis that fruit scent is predictive of sugar content within species. We focus on a single group of chemicals: aliphatic esters. Aliphatic esters are common in many wild fruits [6], as well as domestic cultivars like peaches, apples, bananas, etc. [24–27], and are typically described by humans as having a “fruity” scent [28]. Aliphatic esters are synthesized from alcohols and carboxylic acids via the AAT pathway (alcohol acyltransferase) [29], and the limiting factor to their synthesis is often alcohol [30].This relationship has led to the proposition that the amount of aliphatic esters emitted by fruits should be an honest signal of a fruit’s sugar content since fruit ripening is associated with an increase in alcohols [7, 31], particularly methanol, which is a product of cell-wall degradation, and ethanol, which is the product of sugar fermentation [32–34]. Methyl and ethyl esters—direct products of these alcohols—are also compounds that have been shown to be associated with increased sugar levels across species [18]. Given that all aliphatic esters are synthesized by the same enzymes (AAT has low substrate specificity), it is reasonable to assume that in species that were selected to signal to frugivores via this pathway, increased AAT expression would enhance synthesis of other aliphatic esters as well. Moreover, as opposed to most fruit chemicals, which tend to be biochemically related to those present in unripe fruit, ester emission in ripe fruits shows higher independence from unripe fruits [35], indicating that it may be less susceptible to physiological constraints. Critically, as AAT is synthesized by the plant, emission of esters is not an unavoidable product of fermentation and requires “active participation” from fruit tissue. As such, a positive association between sugar and esters may be interpreted as not only a useful cue for frugivores, but rather an active signal. Finally, frugivorous mammals, and particularly primates, show high olfactory performance with regards to esters [36], indicating that they can be used as a communication channel with seed dispersers. Alternatively, esters have also been found to be expressed in elevated amounts in over-ripe and rotting fruits [37] and in a cohort of fruits of not fully standardized ripeness level be negatively correlated with sugar levels, while primary fermentation products like ethanol are indicative of sugar levels [19].

We first test whether, within species, (I) the total amount (measured as peak area) of aliphatic esters emitted by ripe fruits is positively correlated with sugar level, as opposed to a null hypothesis according to which esters are independent of sugar content. Yet even if present, such an association may not be causal: it is possible that later in the ripening process, fruits are more sugary, and increased metabolic activity in the fruit also emits scent compounds of all kinds, i.e., independent of the biochemical relationship between sugar and esters. To address this issue, we test whether the ester-sugar relationship is not unique and is the result of general upregulation of scent-compound emission by (II) measuring the relationship between non-ester scent compounds (compounds that are not esters and are not synthesized from esters) and sugar, which are likely to be involved in other function like fruit defence [38, 39]. We then test whether (III) the relationship between sugar and esters is limited to esters deriving from ethanol as the main byproduct of fermentation, or (IV) whether it is present also in other esters, indicating that higher sugar levels are associated with increased AAT activity and hence higher total ester emission. Finally, we test whether (V) primary fermentation metabolites—ethanol and its downstream oxidation product acetic acid—predict sugar levels in this species as described for *S. birrea *[19], thus potentially rendering signaling through esters functionally redundant.

**Fig. 1:**
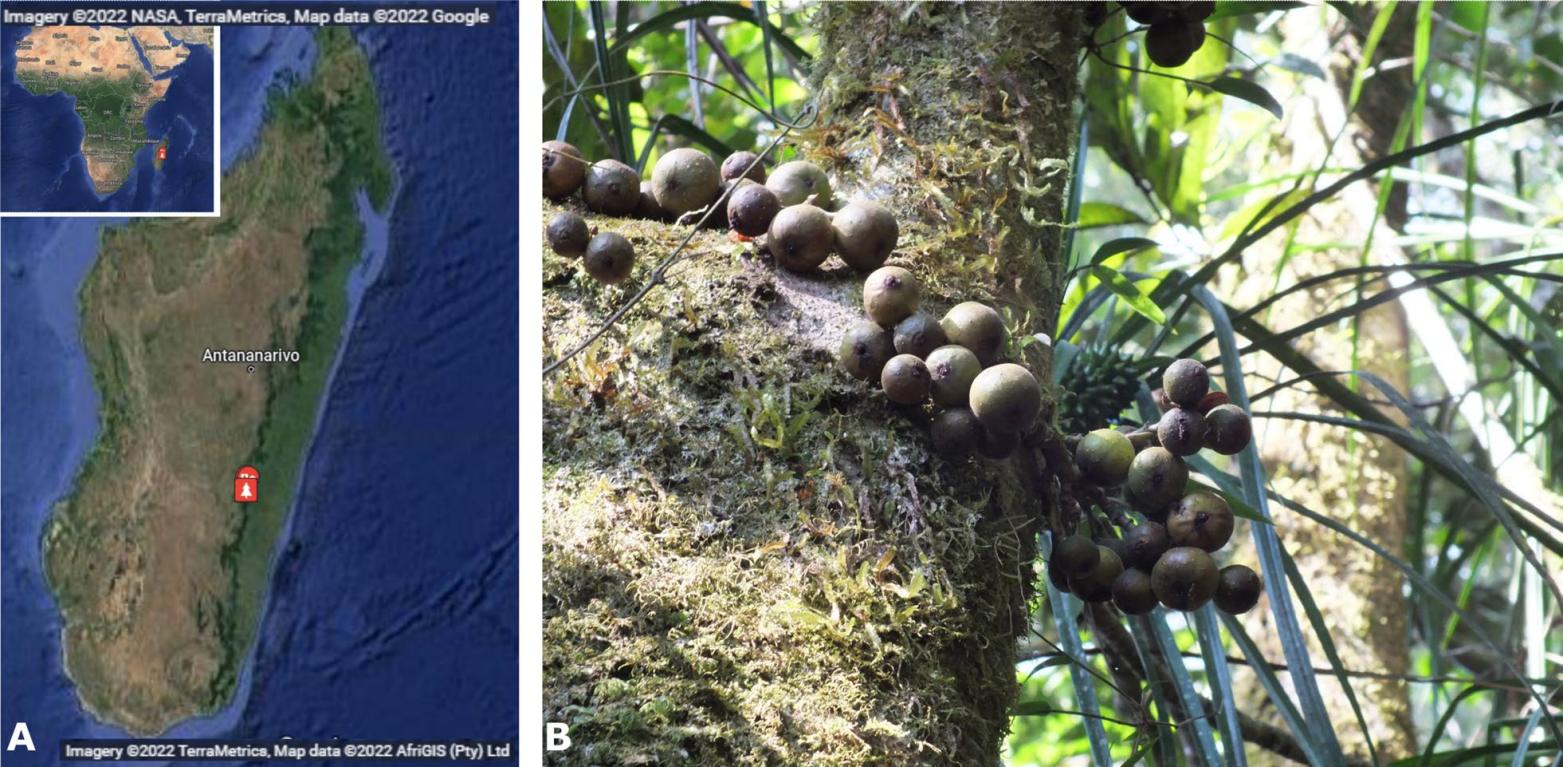
Model system. **A** Location of Ranomafana National Park, east Madagascar. Source: Imagery ©2022 NASA, TerraMetrics, Map Data ©2022 Google; Imagery ©2022, TerraMetrics, Map Data ©2022 AfriGIS (Pty) ltd. **B** *Ficus tiliifolia *in situ (photo: Omer Nevo)

We used *Ficus tiliifolia* (Moraceae) from Ranomafana National Park (east Madagascar) as a model system (Fig. 1). The species is endemic to Madagascar and the Comoro islands [40] and within Madagascar is primarily dispersed by lemurs [41]. Its scent is highly rich in aliphatic esters, and it shows a remarkable shift in ripe fruit scent chemistry from the unripe stage to the ripe stage, indicating that its scent is likely to have evolved as a signal for lemurs [6], thus making it an ideal model species for the questions at hand. In addition, fig trees tend to have high within-tree synchrony in fig maturation, allowing us to sample a cohort of fruit that is at the same ripeness level and eliminate the effect overripe fruits may have on the results (see also discussion). We first test whether individual trees which bear more sugary fruits also tend to have higher ester emissions. Given that variation in fruit quality exists also within trees [42], we then test whether the signal-reward relationship also holds within-tree. The evolutionary logic here is that animals may exert selection on individual plants to advertise fruit quality, and that given the biochemical link between signal and reward in this system this would also manifest in within-individual link between esters and sugars. Moreover, having removed the potential effects of among-individual genetic, developmental, and environmental factors, identification of the sugar-ester link in this level would further strengthen the hypothesis that a biochemical link between signal and reward is present. Using linear and generalized linear mixed models, we show that trees that produce sweeter figs also emit higher amounts of esters and that this relationship is retained even within a single tree. We also find that non-esters are correlated with neither sugar nor esters, showing that the significant correlation between sugar and esters likely represents a genuine biochemical link between signal and reward, with implications for both animal ecology and the evolution of fruit signals in the context of seed dispersal.

## Results

We identified 98 volatile organic compounds (VOCs) in ripe *Ficus tiliifolia* figs (Additional file 1: Table S1). 35 were classified as aliphatic esters (I), of which 20 were directly ethanol derived (III) and 15 were not (IV). We found 63 compounds classified as non-esters (II), including various mono- and sesquiterpenes, alcohols, aldehydes, and aromatics. Two constituents of this category were direct fermentation products (V; ethanol and its product acetic acid). Overall, aliphatic esters dominated the scent bouquet of the species, confirming results from a previous study [6]. All common compounds were present in all trees and most individual figs, while a few rare compounds, usually in small amounts, were unique to individual trees.

### Among individuals

**Fig. 2:**
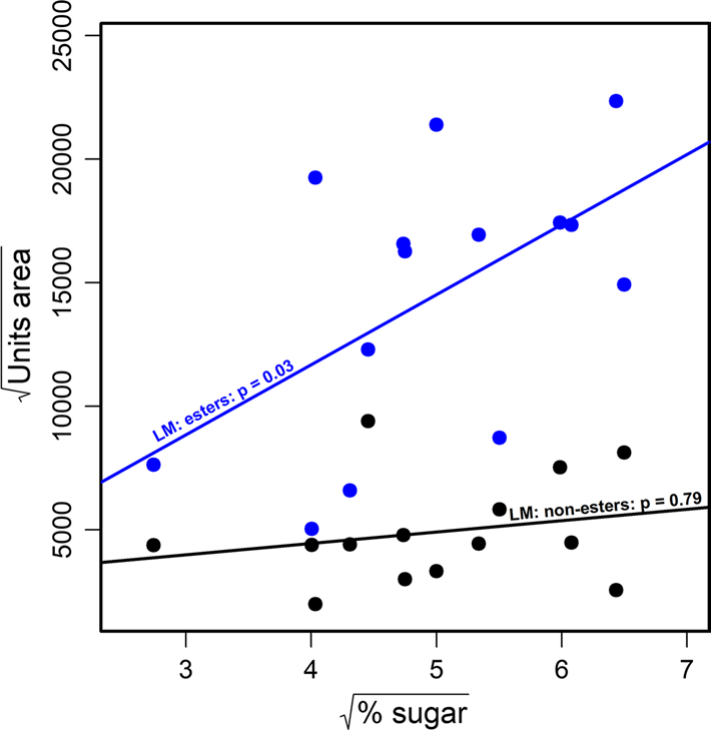
The relationship between percentage sugar and aliphatic esters (I; blue) and non-ester (II; black) scent compounds across individuals. All values are square-root transformed. Results are from linear regression models, where each data point is the average of all figs obtained from a single individual tree, controlling for mean fig dry mass (also square-root transformed). X-axis—% sugar is the relative amount of sugar in dry fig material. Y-axis—“Units area” is the output of the GC–MS (sum of areas under peaks of the relevant group of chemicals) and is a proxy for the total mass of volatile compounds released in a sample

Trees whose figs had on average higher sugar levels (% sugar) also emitted higher amounts of esters (I; linear model, N = 14, t = 2.6(2,11), p = 0.026), and the model as a whole explained about 27% of the variance in sugar (adjusted R^2^ = 0.27). At the same time, the amount of non-esters was independent of sugar levels (II; N = 14, t = -0.27(2,11), p = 0.79; adjusted R^2^ = 0.46) (Fig. 2;). There was a significant positive relationship between sugar and ethanol-derived esters (III; N = 14, t = 2.248(2,11), p = 0.046; adjusted R^2^ = 0.19), as well as other non-ethanol derived esters (IV; N = 14, t = 2.746(2,11), p = 0.019; adjusted R^2^ = 0.46). Finally, primary fermentation products did not correlate with sugar levels (V; N = 14, t = 0.265(2,11), p = 0.796; adjusted R^2^ = 0.35) (Additional file 2: Fig S1).

### Among figs within individuals

**Fig. 3:**
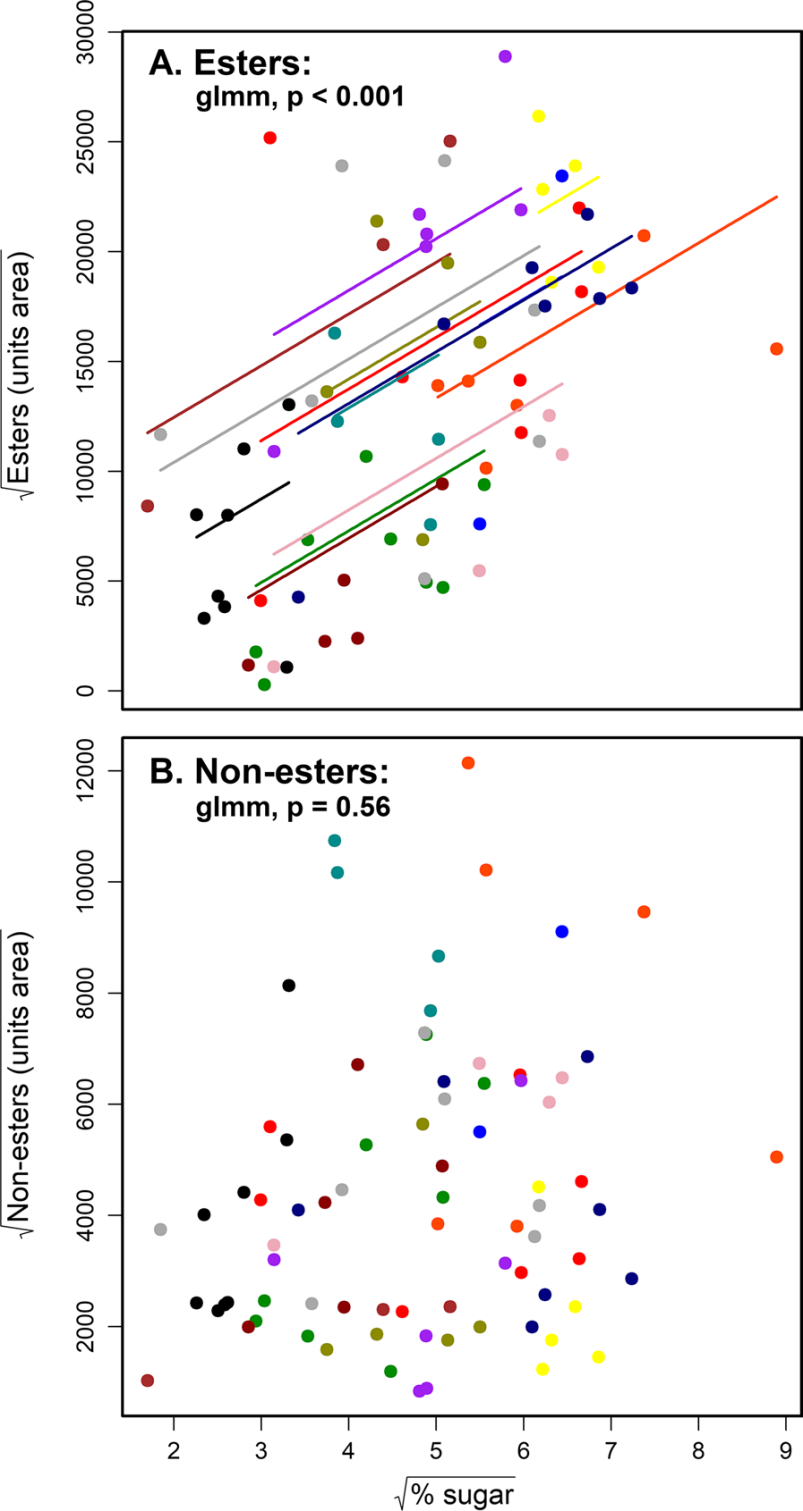
The relationship between percentage sugar and ester (I; **A**) and non-ester (II; **B**) scent compounds of individual figs within individual trees. All values are square-root transformed. Results are from generalized linear mixed effects models with random intercepts, where each data point is the is an individual fig. Colors depict different individual trees. X-axis—% sugar is the relative amount of sugar in dry fig material. Y-axis—“Units area” is the output of the GC–MS (sum of areas under peaks of the relevant group of chemicals) and is a proxy for the total mass amount of volatile compounds released in a sample. The models also included fig dry mass as a control factor to remove the possible effect of fig mass

All relationships amongst variables between trees also held within individual trees. Across the 14 individual trees, individual fruits with higher sugar levels emitted significantly more aliphatic esters (I; likelihood ratio test between full and null GLMMs: χ^2^(1) = 19.3, p < 0.001). The marginal R^2^ (fixed effects only; mR^2^) was 0.24, and the entire model (conditional R^2^; cR^2^) explained 54% of the variance. In contrast, there was no significant association between sugar levels and emission of non-ester volatile compounds (II; χ^2^(1) = 0.34, p < 0.56; mR^2^ = 0.3; cR^2^ = 0.45) (Fig. 3). Ethanol-derived esters were positively associated with sugar levels within trees (III; χ^2^(1) = 18.7, p < 0.001; mR^2^ = 0.23; cR^2^ = 0.56), as did non-ethanol-derived esters (IV; χ^2^(1) = 10.8, p = 0.001; mR^2^ = 0.17; cR^2^ = 0.44). Primary fermentation products (ethanol, acetic acid) did not correlate with sugar levels within trees (V; χ^2^(1) = 0.83, p = 0.36; mR^2^ = 0.23; cR^2^ = 0.34) (Additional file 2: Fig S2).

## Discussion

The main goal of this study was to identify whether the putative biochemical relationship between esters and sugar translates into a potentially useful cue for seed dispersers. Specifically, we asked whether (I) the total amount of aliphatic esters indicates sugar levels in *Ficus tiliifolia*; (II) this relationship holds for non-ester constituents of fig scent; (III) this relationship is driven only by esters deriving directly from ethanol and hence sugar, or alternatively (IV) other esters are also more common in more sugar-rich figs; and finally, whether (V) direct fermentation products (ethanol, acetic acid) are also associated with sugar levels, thus rendering signaling via their esterification redundant.

Our results support the hypothesis that in this model species aliphatic esters signify sugar levels and may be an honest signal for fruit quality. This was apparent both between and within individuals, i.e., trees that tend to produce more sugar-rich figs also tend to emit a higher amount of esters (measured in mass), and within trees, more sugar-rich figs emit a higher amount of esters (I). This is in agreement with the prediction that aliphatic esters, many of them products of sugar fermentation and fruit maturation, would be positively correlated with sugar and may serve as an honest signal of fruit quality [7, 18, 31]. The fact that the relationship is held within trees indicates that the correlation between signal and reward is not only a product of genetic and environmental factors driving variance between individuals, as even when these factors are held constant more sugar translates into higher ester emission.

Our conclusions are strengthened by the fact that non-esters showed no correlation with sugar or esters (II), either between or within individuals. This indicates that the ester-sugar correlation is not merely the result of the fruit maturation process, in which more ripe fruits are simply more odorous and sugar-rich. If this were the case, non-esters would also be emitted in higher volumes when fruits are more ripe and more sugar-rich, and esters and non-esters would be positively correlated. This indicates that only the fraction of volatile compounds whose synthesis is biochemically linked to sugar, i.e., esters, signify sugar levels.

Not surprisingly, aliphatic esters that directly derive from ethanol or its downstream products were positively correlated with sugar (III). This is likely to be the result of a straightforward process: fermentation in more sugar-rich figs leads to more ethanol and its products, which are used for synthesis of volatile aliphatic esters. Yet, aliphatic esters which do not directly derive from ethanol also showed a positive relationship with sugar (IV).This is probably the result of low substrate specificity of AAT [30], the enzyme responsible for ester formation. Presumably, higher sugar levels are associated with higher expression of AAT, which leads to an increase in non-ethanol derived esters as well. The hypothesis that increased sugar levels are associated with increased expression and activity of AAT can be tested in future studies using a transcriptomic approach. Finally, the putative increase in AAT activity is likely to explain the absence of correlation between direct fermentation products (ethanol, acetic acid) and sugar levels (V). Under this scenario, these products are a limiting factor in ester formation, as documented for other species [30], and an elevation in their amounts is quickly translated into an elevation of esters.

While the nominal goal of analyzing both within- and between-trees was to establish the physiological link between signal and reward, it also has downstream implications for animal consumers. The main consumers of *F. tiliifolia* are lemurs, and as primates they are more likely to use spatial knowledge of their habitat to locate fruiting trees while using olfaction primarily for quality assessment of individual fruits once in the tree [43]. While long-distance use of olfaction to find fruit has been experimentally demonstrated in lemurs [44], it is unlikely, although not impossible, that the subtle differences between trees here would be useful for fruit-foraging consumers. This implies that within tree variation is ecologically much more relevant, allowing consumers to quickly assess whether an individual fruit should be ingested or not. It has been shown that the ability to visually assess fruit quality increases fruit intake rates in other primate species [21], a mechanism that may be replicated in cases where olfactory signals provide reliable information about fruit quality. This could in turn translate into preference of species that provide a *within-individual* signal, leading to the pattern identified here.

Our study focused on figs that are neither unripe nor overripe. This demonstrates that even when ripeness level is standardized and no other cues such as hardness/color (unripe) or clear rot (overripe) indicate fruit quality, the VOC profile of *F. tiliifolia* is indicative of sugar content. From this it is likely to predict that signal and reward are also linked to pre- and post-maturation stages. At the same time, in different maturation stages the relationship could differ: in later maturation stages sugar reservoirs may begin to be depleted while their downstream products (esters, in this case) are still being synthesized. This for example is the case in rotting oranges, which tend to emit more aliphatic esters in the rotting process [37]. Yet the decline of the fruit is also associated with an increase in the absolute amount of other metabolites such as ethanol [37]. In this case, it can be predicted that decline is also associated with decreased esterification by the fruit, which would lead to accumulation of ethanol and eventually its downstream product acetic acid. This would in turn strongly alter the *gestalt* of fruit scent, meaning that even higher absolute amounts of esters do not translate into a “sweeter aroma”, i.e., that animals would be able to learn to rely on the sugar-ester relationship identified here during the mature stages of the fruit while using other VOCs to recognize fruits that are over-ripe [45]. Yet these predictions are for future studies to address. Finally, an interesting question that remains open is whether the link between signal and reward identified here is uniformly strong across individuals, or whether a substantial variation in “honesty” among individuals is present. This may be strongly genetically or biochemically wired, but selection pressures, like in many communication systems, may favor different degrees of honesty depending on either environmental or population-level frequency of different phenotypes.

In this context, it is important to note that the link between sugar and esters does not mean that this relationship will hold in any given species or condition. The only other study addressing a similar question found a negative relationship between the major ester constituent, ethyl acetate, and sugar levels in marula fruits in South Africa [19]. In this study, the authors suggested that initial higher sugar levels in a fruit lead to higher ester levels, but that as fruits mature and begin degrading, sugar levels decline and ester levels increase, similar to cultivated citrus fruits infestation by microbes [37]. It could be that the positive relationship identified in the current study is the result of higher synchrony of fruit maturation: in a cohort of fruits that matured around the same time, higher sugar levels would be associated with higher ester levels, whereas a sample of dyssynchronous fruits would include more noise. Indeed, as opposed to many other angiosperms such as *Sclerocaya birrea*, due to their special pollination ecology figs show very high within-tree synchrony [46]. In addition, in species in which ripe fruit scent is dominated by non-ester volatiles, other relationships between signal and reward may play a role, either for sugar or other macronutrients. At the same time, in the absence of a biochemical link between signal and reward, the potential of other chemical classes (e.g. terpenoids, aromatics) to be consistently associated with fruit quality is limited. Finally, since our goal here was to explicitly test the ester-sugar association hypothesis, we treated all other volatile compounds agnostically, i.e., not assuming any ecological role or association with reward quality. Yet it should be acknowledged that some, particularly some alcohols and aldehydes, may also be biochemically related to fruit quality.

Our results add to growing evidence that fruit scent is likely to be an evolved signal to seed dispersers [2]. So far, this has been supported by the notion that in species that rely on seed dispersal by olfaction-oriented animals, fruits tend to change their scent when they become ripe [6, 10, 47]. The inherent biochemical link between signal and reward identified here is the basis on which honest and reliable signals are most likely to evolve [48]. The most likely scenario is that a mild relationship between signal (esters) and reward (sugar) existed in species that began relying on lemurs (or other olfaction-oriented animals) for seed dispersal and that in time their preference for more ester-rich figs exerted selection pressures on figs to increase the ester fraction of their fruit, possibly by increasing AAT expression. This process might also drive the heightened ester emission in overripe fruits [37], or in fruits with overall less sugar [19]: high activity of AAT in fruits that have been selected to advertise their sugar levels via ester production may, as fruits mature, end up advertising this decline in fruit quality. Future studies can test this hypothesis by tracking AAT expression patterns along the fruit maturation process, as well as animal preference.

Interestingly, the suggested increased AAT expression to signal sugar levels to frugivores would create another tradeoff: attracting seed dispersers may require plants to tolerate otherwise damaging microbial activity in fruits to ferment sugars into the precursors used by AAT to synthesize aliphatic esters. This is a classical “two-trait tradeoff” akin to the life-history of the fruit [49]: increased attractiveness would be associated with decreased lifetime. Interestingly, this parallels the defense tradeoff observed in some fruits, in which more attractive fruits in terms of nutrient content tend to be less defended [50]. While governed by a completely different mechanism, the selection regime appears to be similar: lowering defence makes fruits more attractive but more vulnerable, but increased attractiveness can bring faster removal which lowers the fitness cost of potential faster decline.

In summary, using the largest dataset of within-species fruit scent variation, our results show a positive correlation between fig scent profile and reward in a wild fig species, indicating that aliphatic esters may be used as a reliable cue for fig quality. Given the biochemical link between esters and sugar and the fact that esters are actively synthesized by the plant, this relationship is of the sort predicted to be under selection by frugivores [48] and may hence represent an evolved signal rather than only a useful cue. At the same time, these results come from a single model species and from a limited sample size, and only future studies will show to what degree this pattern is representative of other wild fruits. Future studies should also further investigate how target seed dispersers may be using the information coded in fruit scent for fruit selection.

## Conclusion

Fleshy fruits have evolved to attract frugivorous seed dispersers. An increasingly acknowledged mean of communication between fruits and seed dispersers is chemical signaling via fruit scent. It has recently been suggested that aliphatic esters, which are biochemically linked to sugar, may signify sugar content and hence fruit quality, making them potentially honest signals to fruit-eating animals. Our results, focusing on a fig species dispersed by lemurs in Madagascar, support this hypothesis and provide the strongest evidence thus far to the idea that fruit scent contains information on fruit nutrient content.

## Methods

### Sampling

77 ripe figs were collected from 14 *Ficus tiliifolia* trees between 22 Oct and 2 Nov 2018 in the Talatekely area of Ranomafana National Park, eastern Madagascar (Additional file 1: Table S1). At the study site the species can fruit throughout the year, with peaks around June and October–November. Our sampling took place at the beginning of the latter. We sampled figs from all mature *F. tillifolia* trees that produced ripe figs during the sampling period, and sampled all ripe figs to which we had access (2–8 fruits per tree; mean 5.5). Figs were identified as ripe by the typical greenish-yellowish color and softening they present after maturation, as well as the presence of mature seeds. Trees were monitored throughout the sampling period and were collected when identified as mature. This ensured high standardization of ripeness level across the samples. Figs were brought to the lab intact and still on the pedicle. Scent was sampled by enclosing each fig in an unused chamber made of 40 cm of oven bag (Toppits, Germany) which was sealed on one side, and closed around a clean teflon tube on the other. A self made volatile trap made of a 3 cm quartz tube containing 4.5 mg of Tenax TA, Carbotrap, and Carbosieve S-III (all Sigma Aldrich) in equal weights was mounted on the inwards facing end of this tube. The other side of the teflon tube was connected via a silicon tube to a membrane pump. Figs were incubated for 1 h, letting volatiles emitted by the figs concentrate in the chamber. The bag was then emptied onto the volatile trap by pumping its content through the trap at 300 ml/min for 5 min. Traps were stored in teflon-sealed glass vials at − 20 °C until analysis, with the exception of a 24 h transport with ice packs. After odorant sampling, figs were dried in an oven at 40 °C for nutritional analysis.

### Chemical analysis of scent

Samples were analyzed on an Agilent gas chromatograph 7890B equipped with an Agilent DB-Wax polar column (30 m length, 0.25 mm i.d.) and a cold-injection system (Gerstel), coupled with an Agilent mass spectrometer 5977A. Samples were introduced to the thermal desorption unit (TDU) at 3 °C. The liner was set to − 100 °C. After 1 min the TDU began heating at 100 °C until it reached 250 °C. Then, the liner was heated at 12 °C / min until it reached 250 °C, and was held on this temperature for 8 min. Samples were transferred in splitless mode to the column (starting temperature: 40 °C). After 1 min the oven began heating at 10 °C/min until it reached 240 °C, a temperature on which it was held for another 30 min.

Samples were analyzed using Amdis 2.71. Compounds were conservatively identified based on their mass spectra (NIST library 11) and retention index, based on an alkane standard. The identity of 7 compounds constituting on average 82% of the total amount of VOCs (ethanol, methyl acetate, ethyl acetate, methyl butanoate, ethyl butanoate, methyl hexanoate, ethyl hexanoate) was confirmed by injecting analytical standards in the same conditions. We excluded known contaminants (siloxanes, phthalates). We also subtracted the median amount of the respective compounds found in control samples (empty oven bags; 11 controls, sampled daily), to remove compounds that are byproducts of sampling. The resulting dataset (Additional file 1: Table S1) gives an approximation of the total amounts of each compound emitted by the fig during sampling in area units (peak size). This method is suboptimal for estimation of absolute amounts of chemically different compounds since the relationship between peak size and real amount is not consistent across chemical classes. Yet this is likely to introduce only a minor bias in our study since our analyses always compare the amount of the same compounds (e.g. esters) between samples, rather than the amounts of compounds of different classes.

### Nutritional analysis

For analysis of sugar content we calculated % sugar in dry fig material (i.e. excluding water). We used the photometric procedures outlined by Donati et al. [51]. Dried figs were ground to pass a 1 mm sieve and kept in a desiccator prior to analyses. Soluble carbohydrates were extracted with 50% methanol, and soluble sugar concentrations were determined as the equivalent of galactose after acid hydrolyzation of the 50% methanol extract.

### Statistical analysis

Percent sugar, as a proxy for the sweetness/quality of the fruit, was used as the main predictor (independent) variable of variation in scent compounds. We also repeated the analyses using the absolute amount of sugar (%sugar X total dry mass) and received practically identical results (not shown). The response (= dependent) variable in all models depended on the question at hand and was calculated as the sum of the peak areas of the relevant group of chemicals. To test whether total esters are positively correlated with sugar levels (I), we used the total amount of esters emitted by a fig. To test whether non-esters are correlated with sugar levels (II), we used the total amount of all non-esters (i.e. compounds not synthesized by AAT). These compounds primarily include terpenoids and fatty-acid derivatives which are synthesized via other pathways [52]. To test whether the sugar-ester relationship is limited to ethanol-derived compounds (III), we used the absolute amount (measured in peak area) of all esters that either derive directly from ethanol (e.g., ethyl botanoate) or from its oxidation product acetic acid (e.g., butyl acetate). To test whether esters that do not derive from ethanol are also associated with sugar levels (IV), we used all esters not included in the former (= total esters—ethanol-derived esters). To test whether direct fermentation products are associated with sugar levels (V), we used the absolute amounts (peak area) of ethanol and acetic acid. All scent variables are a proxy of the absolute amounts (peak area) emitted by a single fig and measured in peak area units, and are thus comparable only within this study. To control for the possible effect of fig size on scent compound emission, we also included the fig’s dry mass as a control variable. The variables were square-root transformed to approximate a normal distribution. All chemical compounds and their classification with regards to hypotheses I-V are given in Additional file 1: Table S1.

We first tested all five hypotheses (I–V) across individuals, i.e., for example (I) whether on average individuals that produce sweeter figs also emit more esters, but not more other compounds. To test these hypotheses, we calculated the mean values per individual of all variables, and ran linear regression models in which % sugar and dry mass (as a control factor) were the predictors (= independent variables)), and the amount of volatile organic compounds (VOCs) was the response (= dependent) variable.

To test whether these relationships hold within individuals, i.e., whether for example (I) within a tree, sweeter figs emit more esters but not more non-esters, we ran generalized linear mixed models (GLMMs). As above, % sugar and dry mass were the predictor variables and VOCs were the response variables. Individual tree was set as a random intercepts factor, thus assuming that individuals may differ in their “baseline sweetness” due to environmental or genetic factors, but that the relationship between sugar and esters is similar across individuals. It is possible that this assumption is somewhat simplistic and that the strength of the link between sugar and VOCs (slope) varies across individuals, but random-slopes models were unstable and did not provide reliable results (although note that they were qualitatively very similar to the simpler ones reported here). P-values were obtained using a likelihood ratio test comparing the full model to a null model identical to the full model, excluding the main predictor variable (% sugar) but still containing the control variable (dry mass) and random factor. We used the R package MuMIn to extract marginal R^2^s (mR^2^ = 0.3; fixed effects only) and conditional R^2^s (cR^2^; entire model—fixed and random effects).

For all models, we verified the normality and homogeneity of the residuals using histograms, qq-plots, and plotting the fitted vs the residuals. All analyses were conducted on R 3.6.1 [53] and packages lme4 [54], yarrr [55], and MuMIn [56]. Raw data and R code are available as online supplementary materials.

## Supplementary Information

**Additional file 1. **Raw data.

**Additional file 2. **Supplementary figures.

## Data Availability

All raw data used in this manuscript are available in Additional file 1: Table S1. The R code used to obtain the results reported is available in the online supplementary materials.

## Abbreviations

AAT: Alcohol acyltransferase
GC–MS: Gas chromatography mass spectrometry
GLMM: Generalized linear mixed model
TDU: Thermal desorption unit
VOC: Volatile organic compound
