# Supplementary material for: Fruit scent as an honest signal for fruit quality
Source: BMC Ecol Evol. 2022 Nov 30;22:139. doi: 10.1186/s12862-022-02064-z (PMC9710009; doi:10.1186/s12862-022-02064-z)
Supplement: Supplementary file 2 — Additional file 2. Supplementary figures. [file 12862_2022_2064_MOESM2_ESM.pdf]

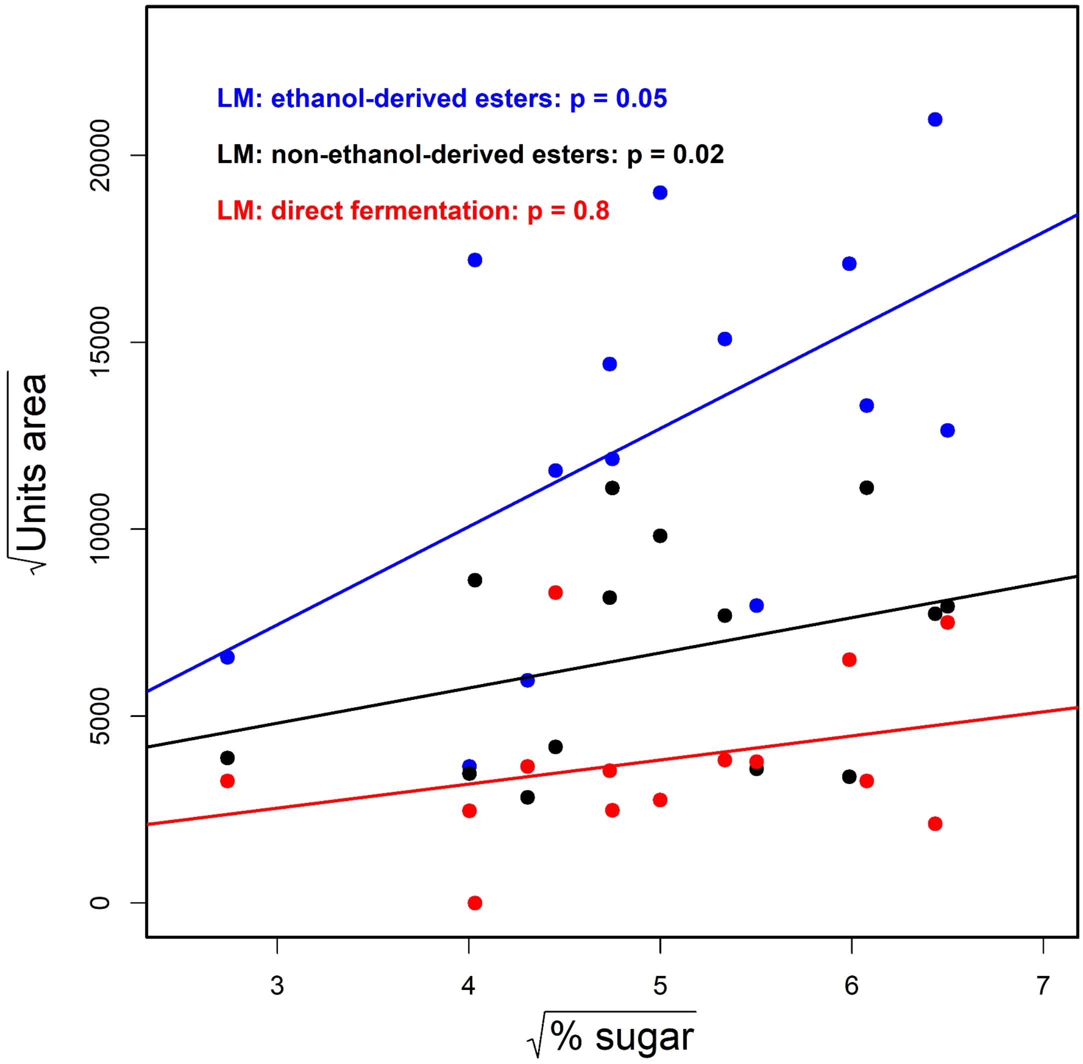

Figure S1. The relationship between percentage sugar and ethanol-derived aliphatic esters (III; blue), non ethanol-derived esters (IV; black), and direct fermentation products (V; red) scent compounds across individuals. All values are square-root transformed. Results are from linear regression models, where each data point is the average of all figs obtained from a single individual tree, controlling for mean fig dry mass (also square-root transformed). X-axis - % sugar is the relative amount of sugar in dry fig material. Y-axis - “Units area” is the output of the GC-MS (area under peaks) and is a proxy for the total mass of volatile compounds released in a sample.

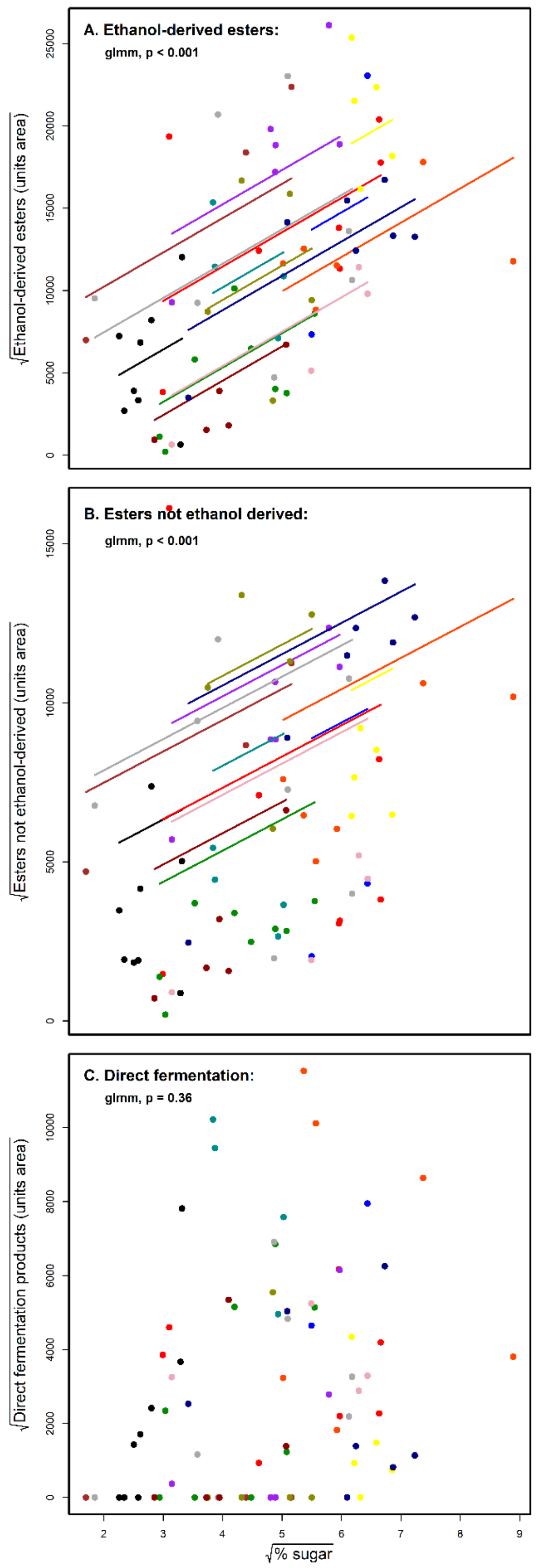
A. Ethanol-derived esters:
glmm, p < 0.001
√Ethanol-derived esters (units area)
B. Esters not ethanol derived:
glmm, p < 0.001
√Esters not ethanol-derived (units area)
C. Direct fermentation:
glmm, p = 0.36
√Direct fermentation products (units area)
√% sugar

Figure S2. The relationship between percentage sugar and ethanol-derived esters (III; A), non-ethanol derived esters (IV; B), and direct fermentation products (V; C) scent compounds of individual figs within individual trees. All values are square-root transformed. Results are from generalized linear mixed effects models with random intercepts, where each data point is the average of all figs obtained from a single tree. Colors depict different individual trees. X-axis - % sugar is the relative amount of sugar in dry fig material. Y-axis - “Units area” is the output of the GC-MS (area under peaks) and is a proxy for the total mass amount of volatile compounds released in a sample. The models also included fig dry mass as a control factor to remove the possible effect of fig mass.
